# Dream Patterns in Patients with Acute Myocardial Infarction: Data from the STEP-IN-AMI Trial

**DOI:** 10.3390/jcm15010231

**Published:** 2025-12-27

**Authors:** Adriana Roncella, Vincenzo Pasceri, Christian Pristipino, Loreta Di Michele, Diego Irini, Robert Allan, Francesco Pelliccia, Giulio Speciale

**Affiliations:** 1 Intensive and Interventional Cardiology Unit, San Filippo Neri Hospital, 00135 Rome, Italy; vpasceri@hotmail.it (V.P.); pristipino.c@gmail.com (C.P.); giulio.speciale@aslroma1.it (G.S.); 2 International Association of Ontopsychology, Via Fontanarosa 25, 00177 Rome, Italy; 3 Institut Coeur Paris Centre, 75004 Paris, France; 4 San Camillo-Forlanini Hospital, 00152 Rome, Italy; dottoressadimichele@gmail.com; 5 Division of Cardiology, Weill Cornell Medicine, New York, NY 10065, USA; cardiacpsych@msn.com; 6 Department of Cardiovascular Sciences, University Sapienza, 00185 Rome, Italy; f.pelliccia@mclink.it

**Keywords:** ontopsychology, dream analysis, ischemic heart disease, acute myocardial infarction

## Abstract

**Background**: Studies on the organization and structure of dreams before and after acute myocardial infarction (AMI) are lacking. **Methods**: We retrospectively studied dream patterns before and after AMI in the STEP-IN-AMI trial (Short-TErm Psychotherapy IN Acute Myocardial Infarction). We also performed an analysis to describe how this pattern may change during ontopsychological short-term psychotherapy (STP) performed after AMI. Forty-seven patients (pts) aged 31–70 were studied. **Results**: At baseline, 21/47 (45%) pts remembered dreams, which increased to 43/47 (91%) with psychotherapy (*p* < 0.0001). Recurring dreams, described as a state of anguish, despair, perceived inability to complete an action, or grief over one’s mother’s early death, occurred before AMI in 16/47 pts (24%). After the third psychotherapy session, no pts reported recurring dreams (*p* < 0.001). In dreams that occurred during the year before AMI, 12 of 25 symbols referred to people known to pts and who had died of a cardiac disease; 9 of 25 symbols referred to an accident, danger, or distressing events. Overall, 21 of 25 symbols were associated with danger to an individual’s life (84%). The incidence of “negative” symbols was sharply reduced during psychotherapy, from 84% to 32% during the first three psychotherapy sessions and to 9% in the last phase of psychotherapy (*p* < 0.0001). **Conclusions**: Our study is the very first on dreams in pts with AMI, and it also examines how STP may change dream patterns in this cohort of pts. AMI pts frequently do not remember dreams that occurred before AMI or report distressing dreams. STP after AMI significantly increased their ability to remember dreams and sharply reduced the incidence of negative/distressing dreams. The results suggest that (1) dream symbols may be connected to the biological status of the dreamer, warning the dreamer of their cardiac condition; (2) ontopsychological STP may act as a stimulus for inner personal change for AMI pts.

## 1. Introduction

Although sleep has often been considered a beneficial period of restoration and well-being, it may significantly impact biological functions [1]. In particular, quality of sleep has been associated with cardiac events [2], and irregular sleep has been associated with subclinical atherosclerosis [3]. Emotional stress represents a trigger for acute coronary syndromes [4], possibly inducing coronary artery spasm [5], activation of the coagulative system with stress-induced thrombogenesis [6], and activation of the neuroendocrine system [7,8]. Sleep complaints have been associated with risk of incident acute myocardial infarction (AMI), and many individuals who have suffered from AMI report sleep disturbances prior to their acute cardiac event [9,10].

Dreams occurring both during rapid eye movement (REM) sleep and non-REM (NREM) sleep may cause sudden variations in sympathetic/parasympathetic balance activity, despite hemodynamic and respiratory stability [1], and may be a source of intense distress. Changes in autonomic activity during dreaming can have negative effects in patients (pts) with cardiovascular disease, as shown in several case reports of pts with no underlying cardiac disease, where emotional stress produced by nightmares or “deadly dreams” led to AMI through spontaneous coronary artery dissection or vasospasm [11].

A wide field of research on dream content and neurophysiology has been developed by cognitive neuroscience. Some studies have been conducted on dreaming in various pathological conditions as schizophrenia [12], pregnant and postpartum mothers [13], and pts suffering from burn pain [14].

However, there are no studies on the organization and structure of dreams before and after myocardial infarction that might convey insight into acute coronary syndromes, pathophysiology, and mechanisms, or on mind/body relationships. The lack of studies on dream characteristics in pts with ischemic heart disease clashes with the wide scientific research that clearly demonstrated the great contribution of psychosocial risk factors (depression, anxiety, acute and chronic stress) to the pathogenesis of atherosclerosis and clinical ischemic events [15,16,17], and the numerous psychotherapy interventions that have been implemented [18].

Therefore, we retrospectively studied dream patterns before and after AMI in the STEP-IN-AMI (Short-TErm Psychotherapy IN Acute Myocardial Infarction) trial [19,20,21]. We also describe how these patterns may change during psychotherapy.

## 2. Materials and Methods

The STEP-IN-AMI trial, approved by the San Filippo Neri Hospital ethics committee on 26 February 2004, number 1/2004 and registered in 7 October 2008 on the ClinicalTrial.gov: NCT00769366 (NIH-National Library of Medicine, National Center for Biotechnology Information), was a randomized study designed to evaluate the efficacy of Short-Term ontopsychological Psychotherapy (STP) in pts with AMI, treated with a percutaneous trans-catheter coronary angioplasty (PTCA).

In total, 101 pts, ≤70 years old, admitted to San Filippo Neri Hospital in Rome for AMI, were randomized to receive or not receive STP in addition to standard contemporary treatment for AMI. AMI was treated in the acute phase with primary or urgent PTCA of the culprit lesion, within 12 h of the onset of an ST elevation-associated AMI (STEMI, primary PTCA) or within 48 h in pts with a non-STEMI (urgent PTCA). The only exclusion criteria were pts’ refusal to participate in this study or inability to attend the psychotherapy sessions. Detailed descriptions of the protocol, methodology, and one- and five-year results have been previously published [19,20,21].

Only pts randomized to STP are included in the present study.

Administration of psycho-active drugs was not part of the protocol; however, in pts already being treated, psychiatric drugs were not discontinued after enrollment. In particular, only one patient (pt) had been in psychiatric follow-up since his twenties.

Psychotherapy was performed by a single skilled and licensed psychotherapist, with the help of clinical staff, psychologists, and nurses. An STP, derived from the ontopsychological method and specifically adapted by the psychotherapist herself to the context of the research in the field of cardiac psychology, was utilized.

The ontopsychological method is a complex and original synthesis derived from psychoanalysis, analytical psychology, and humanistic–existential psychology, as elaborated by Abraham Maslow [22]. With this approach, the human being is considered a complex system consisting of a unity of psyche and body, where anything happening in the body may influence the psyche and vice versa, as demonstrated by several studies in the field of psycho-neuro-endocrine-immunology [23,24,25,26,27].

We report here a very concise synthesis of the STP performed in this group of pts, extracted from other publications [20,28].

*“Psychotherapy was delivered initially in individual and then group sessions over a 6-month period after the incident AMI. Individual meetings focused on personal history, as emotionally lived by the patient, and on understanding basic expressions of the unconscious dimension, through the interpretation of body and oneiric language. The number of individual meetings was tailored to the specific needs and problems of each patient, ranging from 3 to 11 meetings over a 3-month period……. Over the duration of this brief course of treatment, the psychotherapist helps the patient to gain insights and elaborate on conflicts that need to be resolved, as well as on dysfunctional behaviors and interpersonal relationships. After the initial interviews, … the psychotherapist helps the patient to gain insights into his/her body sensations… The psychotherapist guides the patient to acquire full contact with his/her body, starting from the visceral zone, with the help of abdominal breathing and relaxation techniques. In the final phase of the individual meetings and whenever possible, the psychotherapist guides the patient into deeper insights through dream analysis. ……As the psychotherapist helps the patients to contact the central positive nucleus of their unconscious (the “In Se”), their nightmares cease and/or the patients resume remembering dreams related to their real-life problems. This reflects inner changes orchestrated by the patient.*

*The psychotherapeutic work done during the individual sessions is reiterated during group sessions… The aim of all these processes was to stabilize the pathology and promote global well-being within each patient.”*

The one-year and five-year follow-up results of the study, previously published in 2013 and 2019, respectively [20,21], demonstrated a significant statistical improvement in pts treated with STP, compared to the control group, related to depression, quality of life, new hospital admissions, new medical comorbidities, and angina recurrence. An improvement was also noted in major cardiovascular events (MACE), although the results were not statistically significant.

Dream analysis is a fundamental component of STP, but a systematic dream collection had not been planned before this study. Luckily, details of discussions in individual sessions were systematically provided by the psychotherapist in written reports soon after each session. All pts were asked about their dreams during individual sessions.

Group meetings were not recorded, and a lot of valuable material, particularly dream material, was lost and is not part of this analysis.

As the dream material reported by the psychotherapist would be of great interest globally, it was later decided to extract it from the notes, to classify and translate it into English from the original Italian.

Dreams were classified into different lifetime periods:

1. Childhood and adolescence.
2. Adulthood.
3. Dreams in the year before AMI.
4. New dreams that emerged during the psychotherapeutic training. In this section, the dreams have been adjudicated to every single session, from the first one to the tenth session, which was the last one in which a pt referred to a new dream.

While there is no universally agreed-upon classification, we based our age categories on developmental stages commonly used in the public health and pediatric literature. Childhood is generally defined as the period up to 11 years of age, adolescence from 11 or 12 to 18–20 years, and adulthood from age 20 onward. This classification is consistent with the World Health Organization and public health references [29,30].

## 3. Statistical Analysis

Data are presented as the mean ± SD. Continuous variables were compared using the Mann–Whitney U-test, whereas frequencies were compared using Yates’s corrected Chi^2^ test or Fisher’s exact test as appropriate. Repeated measures within a group were assessed with the Wilcoxon rank-sum test or the Friedman test. A *p* value < 0.05 (2-tailed) was considered significant, and 95% confidence intervals (CIs) were reported when possible.

## 4. Results

Of the 54 pts initially randomized to the STP group, 5 refused to continue in this study, resulting in 49 pts in the treatment group. In 2 of the 49 pts, the notes on their dream history were not available, leaving 47 pts available for analysis. Pts were 31 to 70 years old (mean age 55 ± 8); the group included 3 women and 44 men. Four pts completed only the individual sessions but discontinued with the STP before group meetings.

Supplemental Table S1 shows the complete dream material collected, as spontaneously imparted by the 47 pts, which was subdivided into different lifetime periods.

We identified three classes of pts with respect to the ability to remember dreams:

(A) Of the 47 pts, 4 (8%), aged 53 ± 2, could not recall any dreams during their entire lives or during psychotherapy (“no dreamers”).
(B) Of the 47 pts, 22 (47%), aged 55 ± 7, could not remember dreams from childhood up to the AMI, but began to remember dreams during psychotherapy (“light dreamers”).
(C) Of the 47 pts, 21 (45%), aged 55 ± 9, reported dream material related to their life before AMI, as well as dreams during individual psychotherapy (“high dreamers”).

Some pts experienced “dreaming images” during the body relaxation exercises (that induced a state similar to a light sleep) utilized during the psychotherapy sessions. These spontaneous images might be considered of the same origin as nocturnal dream images, and for this reason, they have been considered available for the analysis and reported in Table S1 [31].

The number of patients able to remember dreams increased from 21/47 (45% with 95% confidence interval 31–59%) at baseline to 43/47 (91% with 95% confidence interval 82–98%) with psychotherapy (*p* = 0.000003), corresponding to a difference of 46% (95% CI 30–63%).

**Figure 1:**
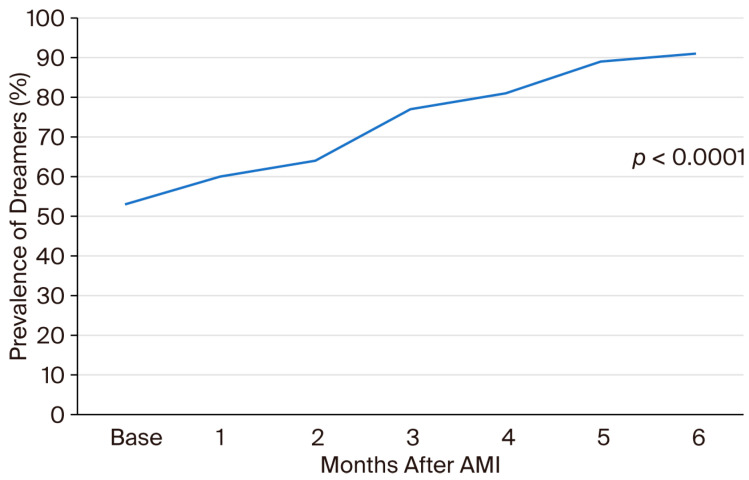
Prevalence of patients able to remember their dreams at the time of AMI and during ontopsychological psychotherapy.

See Supplemental Table S1 and Figure 1 for the temporal trend in patients’ ability to remember dreams from their past and during psychotherapy. There was a progressive increase in patients’ ability to remember dreams during psychotherapy, with a peak at the fifth meeting (Figure 1).

**Figure 2:**
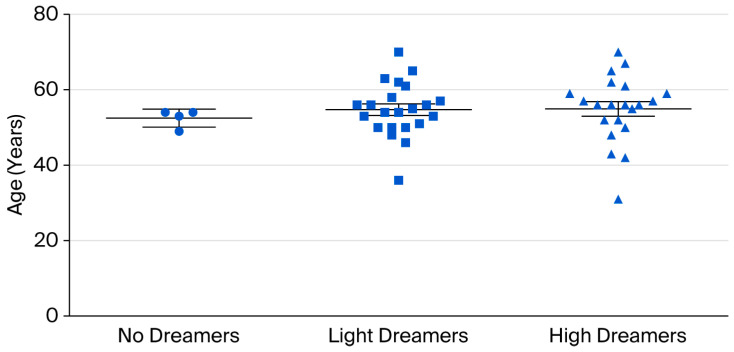
Age distribution of patients according to their ability to recall dreams.

The scatter plot in Figure 2 shows no differences in patients’ ages related to their ability to remember dreams.

Supplemental Table S2 presents a summary of the main symbols that have been extracted from the dreams. Dream symbols included people, animals, inanimate objects, places, landscapes, environments, situations, actions, and states of mind. A column is dedicated to recurrent dreams. This classification has been reported by and modified from Meneghetti A. [31] and Giuliani M. [32].

Recurring dreams in the years before AMI were common (16/47 pts, 34%). No pts had recurring dreams after the third session (*p* < 0.001). Recurrent dreams were characterized by the pts as states of anguish, despair, a perceived inability to complete an action, or grief over one’s mother’s early death (see Supplemental Table S3I(A–F)).

There was little dream content in the reports of the three pts who referred to no-recurring dreams in childhood and adolescence (Table S3II).

Symbols frequently reported in the different periods of life prior to AMI reveal psychological difficulty and conflict (Table S3III).

In the year before AMI (Table S3III(CC)), 12 of the 25 symbols listed referred to people known to the pts and who had died of a cardiac disease; 9 of the 25 symbols referred to an accident, danger, nightmares, and distressing dreams. Overall, 21 of the 25 symbols were associated with danger to the individual’s life (84%). We define these symbols as “negative” because they refer to situations that highlight a threat to the pts’ bio-psychic unity.

For symbols reported during the psychotherapeutic phase, we considered the 47 pts as a whole, who evolved from the first to the last individual session. For this reason, from a psychotherapeutic perspective, we analyzed dreams divided into two phases: a first phase, combining the symbols reported during the first three sessions, and a second phase, combining the symbols referred from the fourth to the last session. This distinction is necessary to allow time for pts to develop inner change.

In the first psychotherapeutic phase (Supplemental Table S4A), the “negative” symbols (in cursive) comprised 26 of the 81 symbols listed (32%). In the second psychotherapeutic phase (Table S4B), the number of “negative” symbols reported in cursive was 14 of 164 (9%). Thus, the incidence of negative symbols was sharply reduced after AMI and during psychotherapy (from 84% to 32% during the first psychotherapeutic phase; *p* < 0.001). They reduced even more to 9% in the second psychotherapeutic phase, with a very high statistical difference compared to the year before AMI (*p* < 0.0001).

Symbols revealing an internal rebirth (in bold in Table S4) of the pts were 32 of 164. They were defined as an “internal rebirth” because they referred to vital situations (young people, children, sunny landscapes, etc.).

The remaining 118 symbols were material classified as useful for clinical analysis (Table S4).

**Figure 3:**
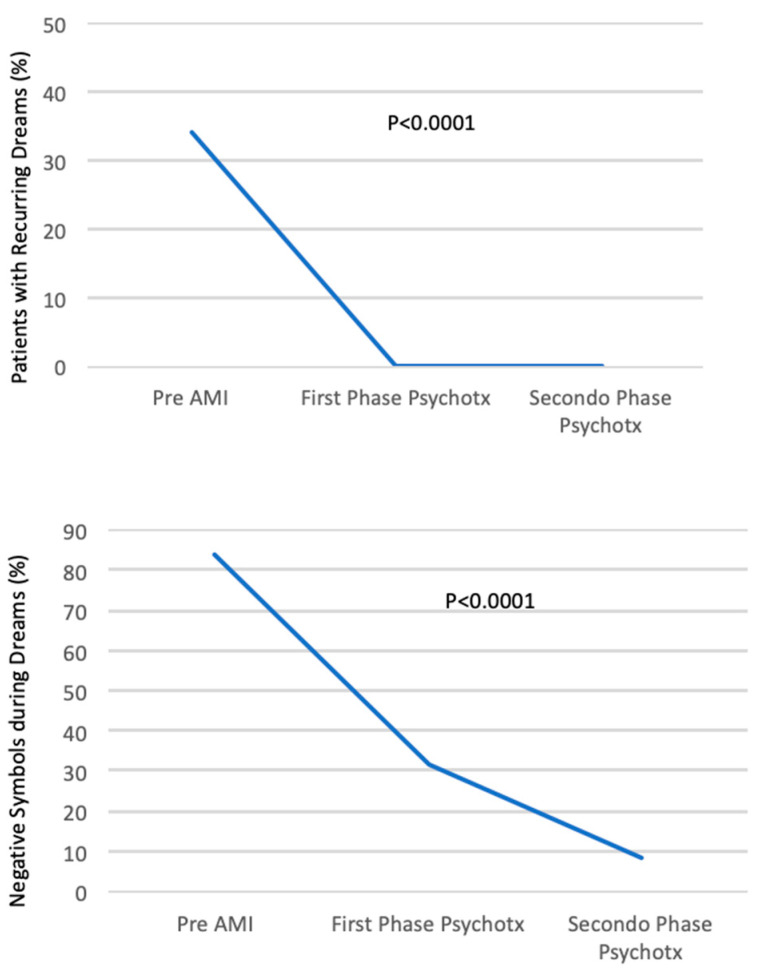

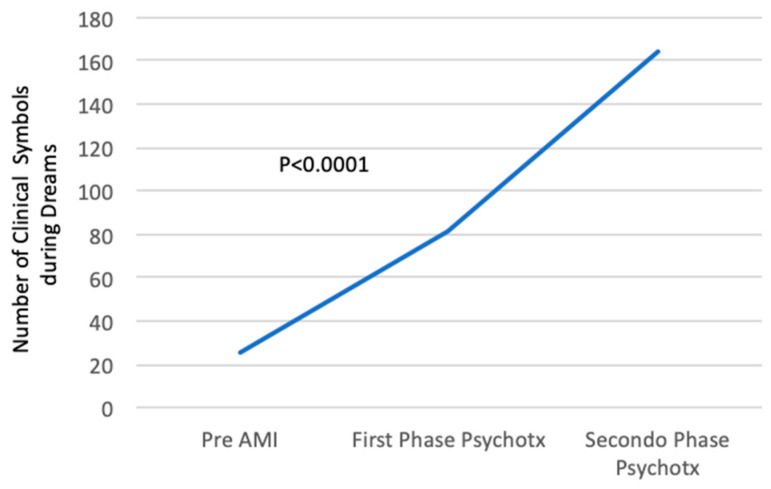
Incidence of recurring dreams, negative dream symbols, and clinical dream symbols before AMI, and their change during the first and second psychotherapy phases.

Figure 3 shows the incidence of recurring dreams, negative dream symbols, and clinical dream symbols before AMI, and their change during the first and second psychotherapeutic phases.

## 5. Discussion

Our study shows that pts with AMI frequently do not remember or report distressing dreams before AMI. STP after AMI significantly increased their ability to remember dreams and sharply reduced the incidence of negative/distressing dreams.

Dream interpretation has been a challenge for philosophers, psychologists, and scientists in every age. However, despite continuous progress in psychotherapy, it is still not clear whether dream analysis could play a role in identifying psychosomatic patterns, particularly in medical conditions.

Sigmund Freud, the founder of psychoanalysis, in his innovative treatise “Dream Interpretation”, published in 1899 [33,34], cites Aristotle’s *De divinatione per somnum* and *De insomniis* [35,36] and Hippocrates’ *Corpus Hippocraticum* [37], which explore possible relationships between dreams and illness. Freud emphasized that an internal bodily stimulus may contribute to dream plot formation [33,34]. He presented a case report from Artigues of a 43-year-old woman who experienced nightmares over many years before her death from cardiac disease. According to Freud, distressing dreams are frequent in patients with heart and lung disease. He cites Tissiè, who affirms that diseased organs confer a characteristic pattern on dream content.

Following Carl Gustav Jung, the founder of analytical psychology, dreams are an integral and fundamental expression of the individual unconscious [38], which communicates by means of symbols. The aim of this communication is represented by integration and self-realization.

Ontopsychology is a recent development in psychodynamic theory. Its perspective on dream analysis is based on the hypothesis that the unconscious is guided by a natural criterion, through which it is possible to distinguish what is useful and functional for the dreamer’s identity. It has been called the *ontic In Itself* [39,40]. The *ontic In Itself* characterizes our specific identity, acting through the vital boost that drives human life, the psychic project throughout one’s life, trying to bring a person to self-realization, in the biological, affective, social, and spiritual dimensions. Following this view, the first step of self-realization is represented by body and psychic health. For this reason, dream symbols are mainly focused on revealing to the subject’s ego the presence of an incipient disease.

If confirmed, from this perspective, dreams might be considered the very first signal of a medical diagnosis and prevention.

This premise is fundamental, because the STP in this group of pts was conducted following the ontopsychological method, which considers dream analysis fundamental to psychotherapy. It must be noted that the present study is not a psychodynamic analysis of dream material collected during STP of AMI pts, but it highlights some unexplored characteristics of this patient population that emerged during psychotherapy.

The first observation that emerged is the high percentage of pts not remembering dreams before AMI (55%). Sleep medicine has shown a close association between rapid eye movement (REM) sleep and dreaming [41], suggesting that every person dreams during sleep. The reason why one does not remember dreams remains an open question. One cause of not remembering dreams may be the use of psychotropic drugs [42], but in this cohort, only one pt was taking psychiatric drugs, and no-one was given hypnotherapy treatment; moreover, the one pt taking psychiatric drugs since adolescence did not remember past dreams but did remember dreams during psychotherapy.

In other studies, it has been hypothesized that age issues are essential for dreaming recall, related to brain sleep maturation, from fetal development to early childhood, going through adulthood and elderly [43]. However, in our study, there was no difference in age associated with the ability to recall dreams, possibly due to the narrow age range of our pts (most pts were between 50 and 70 years, with no pts over 70).

Neurobiology studies have been conducted on dream recall frequency in psychosomatics [44]. Various hypothesis have been advanced. Mangiaruga et al. hypothesized that the ability to dream and remember dreams is associated with the development of cognitive processes (perception, learning, language, thinking, attention, memory, motivation and emotion) [43]. Other neurobiological studies focused on neuroanatomic changes responsible for dream memorization.

The adult limbic system is not static but can be reshaped by experience: glycocorticoids produced during prolonged stress can cause dendritic regression by inhibiting the learning and memorization processes that take place through the activation of the hippocampus [45,46]. Other studies have shown a higher ability to remember dreams upon awakening in people with an increased density of white matter in the medial prefrontal cortex compared to people with low ability of memorization [47]. Lewin et al. [48] observed that in mice the traumas suffered in early developmental stages are associated with sleep alterations that persist throughout the whole life. If this finding also applies to human beings, it could explain the association between sleep disorders and psychosomatic diseases, since the latter, in many cases, seem to develop in the human being as a defense against traumatic events occurring in the very early stages of development.

From a psychological point of view, not remembering dreams may be an expression of censure and psychic repression caused by a strict education in infancy.

For these reasons, we can hypothesize that “not remembering dreams” may have a clinical significance here, and from a psychodynamic point of view, it seems that a lack of awareness of the individual related to their interior personal life, such as progressive psychic repression, starts very early in life. This observation is coherent with the experimental data of neurobiological research, and the finding of a high incidence of “alexithimia” in cardiac pts [49]. Alexithymia literally means “no words for emotions” and refers to a condition in mental health settings when people have difficulties in identifying and verbalizing their emotional states. This mechanism reinforces the view that these pts do not have a complete consciousness of their states of mind and have acquired the habit of continuously converting their emotions into a psychosomatic reaction, in agreement with what is confirmed by psychosomatic medicine [50,51].

Another frequent dream pattern in this cohort is recurrent dreams in the life period before AMI. In total, 16/47 pts (34%) reported recurrent dreams before AMI, referred to as nightmares or a state of anguish, despair, perceived inability to complete an action, or grief over one’s mother’s early death.

Although nightmares may be caused by psychiatric drugs [52], only one pt was taking them, and he did not report nightmares.

Nightmares are frequent in posttraumatic stress disorder (PTSD), and may be referred also by idiopathic nightmare sufferers and healthy individuals [53]. Posttraumatic nightmare sufferers exhibit more nocturnal awakenings than idiopathic nightmare sufferers and healthy subjects, which supports the hypothesis of hyperarousal in sleep in PTSD.

Other studies agree that chronic nightmares develop through the interaction of elevated hyperarousal and impaired fear extinction. This interplay is assumed to be facilitated by trait affect distress elicited by traumatic experiences, early childhood adversity and trait susceptibility [54].

Excessive arousals caused by sleep disorders decreases parasympathetic and increases sympathetic tone. These mechanisms may be associated with adverse effects on cardiovascular health, such as coronary artery disease [55]. Acute risk factors, such as heavy physical activity, emotional stress, eating, cold or heat exposure, coffee or alcohol consumption, cocaine or marijuana use and sexual intercourse, can also suddenly and transiently increase the risk of acute cardiac diseases; it is likely that they act through a sympathetic arousal [56]. These mechanisms show a similarity with those highlighted in PTSD.

From a psychodynamic perspective, the occurrence of nightmares could be considered an alarm signal for a disturbance of current psychic equilibrium [57].

According to this hypothesis, if we analyze here nightmares and recurrent dreams from a psychodynamic perspective, they may represent an attempt by the unconscious to signal a problem to the person’s ego, a problem or conflict that might compromise the harmonic body and psychic evolution of the individual. In fact, the recurrent dreams reported extensively in Table S3 highlight a difficulty, an inability to overcome an obstacle or to reach a goal, a sense of anguish, a danger, or the need to pass an exam. In only a few cases, there was an attempt at sublimation or an escape from life’s difficulties (Table S3A3,A6,B7).

If we evaluate the main symbols that emerged in infancy and childhood (Table S3IA,IIIAA), they reinforce the interpretation of difficulties in personal growth and evolution emerging very early in life.

Symbols seem to emerge here like knots of a net delineating the common foundation of a collective unconscious, in a very similar and repetitive psychodynamic mechanism.

In the year before AMI, the scenarios became worse than before. In particular, 12 of the 25 symbols listed referred to people known to the pts who had died of a cardiac disease; 9 of the 25 symbols related to an accident, danger, nightmares, and distressing dreams (Table S3III(CC)). Overall, 21 of the 25 symbols (84%) highlight danger to the individual’s life. We have defined these symbols as “negative” because they refer to situations that highlight a threat to the pt’s bio-psychic unity: they seem to warn of a danger of cardiac death.

The relationship between cardiac death warning symbols and incident AMI may confirm the ontopsychological hypothesis that dream symbols might be related to the biological status of the dreamer, warning months in advance of an impending cardiac event.

During psychotherapy, dream patterns change, and there is a statistically significant difference for all three patterns described.

The number of pts able to remember dreams increased from 25/47 (53%) at baseline to 43/47 (91%) with a statistically significant difference, as if psychotherapy had acted as a stimulus for inner insight. This may also be considered as a sign of trust and open-mindedness toward the psychotherapist.

After the third session, recurring dreams stopped completely, with a high statistical difference compared to recurrent dreams in the years before infarction. Moreover, during psychotherapy, there was a change in reported symbols. In particular, the incidence of “negative” symbols was sharply reduced after AMI (Figure 3).

During psychotherapy, there were some “positive” symbols of natural landscapes, or young people, or wild animals. They seem to reflect an internal rebirth in some patients. Other symbols reported during psychotherapy show a wider scenario, connected to pts’ real lives, useful for clinical analysis (Figure 3).

The cessation of recurrent distressing dreams and the reduction in the incidence of “negative” symbols during psychotherapy strengthen the hypothesis that psychotherapy acted as a positive stimulus for personal change. This psychological “positive” change might also be supported by results previously published in the STEP-IN-AMI trial [20,21], which showed statistically significant psychological and clinical improvement in the PTS group compared to the control group.

It would be fascinating to explore which neurobiological changes are implicated in psychological and clinical improvement consequent to a psychotherapeutic treatment [58], and which neurobiological phenomenon were correlated to the change in dream patterns in this group of pts [59].

This is the first study that attempts to analyze dream patterns in AMI pts. Moreover, thanks to ontopsychology, which has introduced the interpretation of symbols based on biological meaning, it offers great novelty in psychotherapy research, and it may favor a deep integration between medicine and psychotherapy.

### Limitations

The randomized STEP-IN-AMI trial aimed to evaluate the efficacy of an ontopsychological STP in AMI pts. Dream data systematic collection was not planned in advance but was performed retrospectively.

Dreams reported during group meetings were not recorded, thus possibly missing interesting material and dreams reported by pts. However, this did not influence our data on the dreams occurring before AMI.

Due to the low number of pts, the collected dream data may not be representative of the general cardiac pts population. Moreover, due to the low number of female pts, it was not possible to perform a qualitative and quantitative analysis of dream patterns between the sexes.

The classification reported is extracted from hundreds of individual psychotherapy sessions conducted in the STEP-IN-AMI trial, which utilized an ontopsychological methodology. Consequently, symbol analysis reflects the methodology used.

Dream analysis is a complex task that can only be performed in a psychotherapeutic setting and based on a comprehensive knowledge of a patient’s history. Despite this limitation, we have extracted specific symbols to better analyze the similarities in dream patterns in this cohort of pts. However, it must be emphasized that our results cannot be directly extrapolated to other psychotherapeutic strategies.

The stress axis, cortisol secretion, or other neurobiological evaluations might be important in this group of pts for explaining the production of specific dream content [4] were not scheduled in the trial protocol. They might be added in future research. In particular, neurobiological studies might shed light on biological phenomena correlated with dream patterns in AMI pts, and how they can change during psychotherapy.

Further studies are needed to evaluate dreams in a larger cohort of AMI pts, to compare our results with a healthy subject control group. For this reason, a new research protocol is ongoing: the PSYCHONIC (PSYChosomatic medicine in ONcologIc and Cardiac disease) trial [60].

## 6. Conclusions

On the basis of these results, we hypothesize the following: (1) the high percentage of pts not remembering dreams before AMI may be an expression of censure and psychic repression; (2) the high percentage of symbols of people who died of a cardiac disease and symbols of an accident, danger, nightmares, and distressing dreams in the year before AMI might indicate that dreams are connected to the biological status of the dreamer, warning the dreamer of their cardiac condition, coherent with the ontopsychological theory; (3) the progressive increase in the ability of the pts to remember dreams, the cessation of recurrent distressing dreams, and the reduced incidence of “negative” symbols during psychotherapy strongly suggest that psychotherapy acted as a positive stimulus for inner personal change; and (4) if these data are confirmed in other research, dream analysis might become a fundamental tool to integrate into psychological intervention and rehabilitation of AMI pts.

## Data Availability

All the data related to the STEP IN AMI trial are available in the paper archives of the study. The data utilized for the statistical analysis are available in an Excel datasheet.

## Supplementary Materials

The following supporting information can be downloaded at: https://www.mdpi.com/article/10.3390/jcm15010231/s1.
