# Supplementary material for: Dream Patterns in Patients with Acute Myocardial Infarction: Data from the STEP-IN-AMI Trial
Source: J Clin Med. 2025 Dec 27;15(1):231. doi: 10.3390/jcm15010231 (PMC12786656; doi:10.3390/jcm15010231)
Supplement: Supplementary file 1 [file jcm-15-00231-s001.zip › Table S2.pdf]

TABLE S2. ANALYTIC CLASSIFICATION OF DREAM SYMBOLS

SYMBOLS	Dreams of childhood and adolescence	Dreams of adult life	Dreams in the year before AMI	First individual session	Second individual session	Third individual session	Fourth individual session	Fifth individual session	Sixth individual session	Seventh individual session	Eight individual session	Ninth individual session	Tenth individual session
People	1) Dead mother. 2) Dead mother.<br>3) Mother	1) My brother 2) my dead wife with a vague face	1) My father and mother (who were dead in real life) 2) my dead father 3) my grandmother who had died for a liver disease 4) my mother in law, dead when she was 86 years old for a cardiac disease 5) the father of my wife's actual partner, who had died for a cardiac disease 6) my son 7) father in law, dead for sudden death		1) My daughter<br>2) A very nice and open-minded girl 3) My two children 4) Two longstanding friends of mine	1) My brother<br>2) My mother in law, dead 10 years ago<br>3) A friend of mine, who is an accountant<br>4) My son's godfather. He was going bankrupt<br>5) My colleagues and my friends<br>6) My wife was nude<br>7) My brother<br>8) My mother<br>9) My ex building site boss<br>10) A small very beautiful child<br>11) A little 8 year girl, who is my daughter	1) My 83 year old mother, who is a cardiac patient, already operated on of a coronary artery bypass grafting<br>2) Pope<br>3) A beautiful half-naked girl<br>4) My brother<br>5) My children<br>6) My head clerk of two years ago<br>7) A film director, who had died when he was 59 years old<br>8) My friends of Milan<br>9) My second daughter's partner, who was arrested by the police<br>10) My first daughter	1) Some friends<br>2) My little grandson<br>3) My next-door shop neighbor, who was dead for a tumor<br>4) A very tall and robust build friend of mine<br>5) A person, who has a debt with me of 350 thousands euros<br>6) My father, all black, like a mummy, without eyes<br>7) My 38 year old nephew<br>8) A lady, a Cardiologist's wife, whom I had had a long love affair with about 10 years ago<br>9) My friend Claudia<br>10) My father was alive (in real life he died 10 years ago)<br>11) My brother<br>12) My wife	1) My father's brother, who was a delinquent and had been imprisoned in real life, whereas in the dream he was dressed as a policeman<br>2) My daughter<br>3) My family female doctor<br>4) My family female doctor's 2 year old daughter<br>5) A woman with fishnet tights, that are similar to the net put around the roast beef	1) My wife<br>2) A serviceman<br>3) A person who keeps a brown dog on a tight rein<br>4) A little girl<br>5) A colleague of mine<br>6) My dead grandmother<br>7) My brother			
Animals						1) A dog.	1) Dogs<br>2) Animals in a farm (a cow and others)<br>3) Wild boars		1) A herd of multicolored horses in the paddock	1) Dogs			
Non animated objects	1) Moped				1) A photo<br>2) Knives<br>3) Cigarettes<br>4) Cigarettes	1) A motorcycle	1) Bookcase	1) My car<br>2) My car<br>3) A sailing boat, all black<br>4) Some small tables and chairs, all empty	1) A beautiful church with some icons<br>2) A kind of pedalo with oars, that is dismantled<br>3) A house<br>4) A fireplace<br>5) A locomotive<br>6) The container of my two credit cards, that was empty	1) Graves with tombstones of typical from Lecce stone<br>2) Many monitors with electric wires<br>3) Staircase<br>4) Car<br>5) Car	1) Ship		1) A stair<br>2) Water<br>3) Little stones.
Places	1) Square 2) mud 3) basement 4) my house	1) School. 2) square 3) I am on the big and wide staircase of my secondary school 4) big old building near Venezia square in Rome 5) important and severe rooms 6) lower secondary school	1) Square		1) School	1) I was with other Italian people at Bracciano (the town where I live)<br>2) I am with my wife in intimacy in our bedroom<br>3) A castle<br>4) A cemetery<br>5) My working place	1) My house	1) I was at Anguillara (my town that is on a lake)<br>2) A cemetery completely flooded<br>3) Bar<br>4) A port<br>5) The railway station<br>6) A shaky staircase<br>7) A courtyard with a big walnut tree	1) I was in Belgium	1) Tunnel<br>2) Castle<br>3) Cemetery	1) Ship		1) Garage
Landscapes	1) Sea	1) Sea	1) Sea	1) I was on a reef; in front of me there was the sea, that was like a pond	1) Lake shore		1) Desert	1) A dark blue sea, all the landscape is dark blue<br>2) Greenery, there is a small lake<br>3) It is dark	1) Stormy sea<br>2) A starry sky<br>3) A steep slope, deep and rumbling gorge; around there were many trees, a chestnut tree, fir trees; in the middle of the trees some ruins<br>4) I am with the animals of my shop on the see shore<br>5) Some islets remain on a blu and calm sea<br>6) I was in a green valley<br>7) The sky was blue	1) A ravine<br>2) A green valley<br>3) The Maldive islands landscape, the blue sea<br>4) Many meadows and green places,	1) The Alps		
Environment	1) Half-dark room. 2) very rich and colored dreams		1) it was raining			1) Everything was dark<br>2) A cemetery; it is dark							
Situations	1) Nightmare 2) danger coming from unlikely beings, who might grasp me, catch me. 3) I felt myself grasped by these unlikely beings	1) Very distressing dreams, and sometimes nightmares. 2) military service 3) nightmare 4) beautiful, relaxing and radiant dreams 5) nightmares	1) Distressing dreams 2) nightmares	1) Nightmares.	1) Very distressing dreams and nightmares. 2) I am feeling my body 3) I was dressed with a jeans suit 4) I am a Rome supporter	1) I was into a castle; there was a female presence 2) Nightmare	1) I lied on the bed with my mother, who is 83 years old. She is a cardiac patient<br>2) I was a young man, and I was with a film director, who I had worked with some years ago for a film shotted in the desert. In real life this director had died when he was 59 years old<br>3) My job	1) I and my father in a port on a big sailing boat, that was all black<br>2) I was at the railway station; there was much crowd<br>3) I am on a shaky staircase, that I must climb to reach the attic room	1) A flash of colored lights		1) I am on a ship; there are many tidy rooms, things to eat		1) I am in a garage. There is a stair, that goes into the garage
Actions	1) I follow a downhill street that arrives to the sea. 2) I can see the sea, but I never arrive to touch it. 3) I was sinking in the mud. 4) I remained in the half-dark room of the basement. 5) with a big effort, I was struggling to free myself, and I woke up 6) to go on a moped. 7) I was living at the last floor of a building. I jumped from the balcony; then, when I arrived to the lower floors, I circled and I landed slowly. 8) I climbed again the stairs and rang the bell, and my mother opened the door.	1) I had to take my exams again.<br>2) speaking in a very harrowing way. 3) I follow a downhill street that arrives to the sea. 4) I can see the sea, but I never arrive to touch it. 5) to do military service 6) to loose 2 years of one's life 7) to climb on a grassy slope 8) stones rolling down against me 9) I enter in a small door 10) to go toward a dark and variegated room, that is becoming bigger and bigger, huge 11) I try to defend myself inside a fortress or over a hill 12) to fall down 13) I was driving my car and I had an accident" A tsunami is sweeping away and destroying everything 14) I was a young boy at the lower secondary school 15) I couldn't succeed to pass my exams 16) flying	1) I follow a downhill street that arrives to the sea. 2) I can see the sea, but I never arrive to touch it. 3) I had lost my car 4) My father and mother (who were dead in real life) following me 4) to be run over by a tsunami 5) I sow myself reflected in a mirror, and there was the image of my dead father 6) I was falling down an abyss 7) falling off while I was sleeping 8) my grandmother, dead for liver disease, was very fat, and we were having lunch with many relatives 9) my mother in law, dead for a cardiac disease, entering in my house and giving me her hand 10) I accept and I give my hand to my mother in law, dead for a cardiac disease 11) My son called me to tell me that he had had a car accident 12) father in law, dead for sudden death, invited me to go with him, telling that beyond one can stay very well	1) A scaffolding falling on top of me. 2) A hand pushed me and I fell in the water. I went to the bottom and I couldn't go up. I felt the water weight, and I felt that I was drowning	1) I have to take my exams again. 2) A photo taken by my daughter 3) I have seen a shadow, that is my own image, watching at me from all the sides. 4) I sow knives 5) I was playing football in my house garden with two longstanding friends of mine 6) I attend a game between Rome and Juventus (two Italian football teams) 7) The Juventus team wins the game 8) To smoke a sigarette 9) I had to complete a work in the building site, where I am working 10) I was smoking a pack of cigarettes	1) I was speaking very calmly with my brother 2) We left with a big ship to go to work in America, manual jobs as carpentry, etc.<br>3) We can revolutionize the American society 4) We come back to Italy<br>5) My son is passing to go to the bathroom and sees me and my wife in intimacy through the door<br>6) I was going back to my work, and I found again my colleagues and my friends; it was a positive situation<br>7) I was dressed for a hunting party, but I couldn't see the place, and everything was dark<br>8) I couldn't go out of the castle<br>9) My wife was nude and was rubbing cream into her previous employer's penis<br>10) I gave three strong slaps in my wife's bottom<br>11) Smoke is coming out from a grave<br>12) My father's body is burning. I extinguish the fire and try to recompose my father's body<br>13) I was with my brother, and we had a colored bandanna on our head. We were cooking; my brother was cooking the first course , whereas I was cooking barbecued meat<br>14) My mother, who was helping us with the two children<br>15) A tree fell down on my bed<br>16) I took a small child in my arms<br>17) Situations where I cannot reach a goal<br>18) I am driving a motorcycle toward a high hill<br>19) The motorcycle broke and stopped<br>20) I try to repair a wire that goes to the carburetor, without success<br>21) I say to my daughter that we have to send away the dog, and I am sorry	1) I was running away, chased by someone that I couldn't identify<br>2) My 83 year old mother was giving off a yellow liquid from her mouth<br>3) I sow Pope Giovanni Paolo the 2nd<br>4) Pope Giovanni Paolo the 2nd was giving his hand to me<br>5) I kissed. a beautiful half-naked girl all up to her pubis<br>6) I was working on a scaffolding<br>7) I slipped and risked to fall down in an empty space<br>8) I called out loudly and asked for help to my brother, who ran to help me<br>9) Some little dogs arrive to my house<br>10) my head clerk of two years ago says to me to give something to eat to the animals in a farm (a cow and others), but I forget to do this<br>11) I couldn't have the hunting license<br>12) There were many wild boars, and I couldn't shoot anyone<br>13) I threw books on a person, but I cannot say who that person was<br>14) The policeman showed me the identity card of the arrested boy (my son in low), and there was written 'ROM'. I answered that I was right, he was a ROM, a gypsy	1) Some friends, that I couldn't see, threw a closed mussels necklace to me<br>2) My little grandson taken in his mother's arms<br>3) I want to do more in the workplace, often replacing other colleagues, also when they do not desire this, and trying to obtain a social consensus<br>4) I can save myself climbing on the roof of a car, in a cemetery completely flooded<br>5) I am walking on a road going uphill, but I am not so courageous to look further on<br>6) I was going to a bar and I met a friend of mine<br>7) My friend accused me of something, and I defended myself<br>8) I went out of the bar, and I couldn't find my car<br>9) I am driving my car in the traffic<br>10) Cars coming from the opposite direction<br>11) I sow my father, all black, as a mummy without eyes<br>12) My father, with empty eyes, turns toward me and says to me that we have to start a new refreshment place<br>13) I was looking for my 38 year old nephew, and I found him<br>14) I am walking with you (the psychotherapist) through greenery<br>15) I kiss you (the psychotherapist)<br>15) I hold a child's hand and I arrive into the attic room with hard physical exertion<br>16) I, Claudia and the kid leant against the balcony railing, that breaks, and we fall down in the courtyard<br>17) We hold on to the broken railing, and we succeed in falling down on our feet without hurting ourselves<br>18) My father was alive (in real life he died 10 years ago), and he was coming with my brother and offered me a lot of things to eat<br>19) I was trying to transfer some decisions to a collaborator of mine<br>20) My collaborator answered me that it was okay if he had already decided<br>21) I go to a fair with my wife<br>22) My wife goes away with a male doll	1) I assembled a dismantled pedalo<br>2) I started rowing on a stormy sea<br>3) I was on a locomotive, that was running fast through a steep slope, deep and rumbling gorge<br>4) A wave arrives and swamped everything<br>5) My daughter was taking a coca-cola from the refrigerator<br>6) I told my daughter to drink all the coca-cola can<br>7) I am looking a brown running horse, that is ridden by my family female doctor, who is turned on the contrary, toward the bottom of the horse, and she holds in front of herself her 2 year old daughter<br>8) I put a basil bunch under the net of fishnet tights of a woman	1) I dreamt that my wife was telling me what she had dreamt, and I was telling her what I had dreamt<br>2) I am talking to a serviceman, and I say to him that 120 days have passed, and he answers: 'yes 4 months'<br>3) I meet a person who keeps a brown dog on a tight rein.<br>4) A dog coming along the street tries to attack me.<br>5) I find a pillow and I try to defend myself from the dog<br>6) The dog attacks me again and grabs me with his leg across the pillow<br>7) A little girl, who cries in despair<br>8) A colleague of mine and I wander around Rome, and we enter in a cemetery<br>9) I cannot eat food<br>10) We go out by a car, and my friend is driving<br>11) I was driving a car on a bend mountain street<br>12) I start flying on a green valley<br>13) My brother is guiding me<br>14) I enter in a tunnel<br>15) I emerge into a castle<br>16) I get always in a street, that takes to a ravine			1) I have to tidy up the entrance of the garage, so that water cannot enter.
State of mind	1) Desperate anguish	1) I was anxious that I wasn't up to it"					1) My children are happy<br>2) I felt anguish	1) There is peace<br>2) I am afraid of being disrespectful to you					
Recurrent Dreams	6 patients	7 patients	3 patients	1 patients	2 patients	1 patient							

Childhood: period from infancy to the age of 11.
Adolescence: period approximately from the age of 12 to 20.
Adult life: period from the age of 21 to the beginning of the year before AMI
AMI: acute myocardial infarction
