# Supplementary material for: Dream Patterns in Patients with Acute Myocardial Infarction: Data from the STEP-IN-AMI Trial
Source: J Clin Med. 2025 Dec 27;15(1):231. doi: 10.3390/jcm15010231 (PMC12786656; doi:10.3390/jcm15010231)
Supplement: Supplementary file 1 [file jcm-15-00231-s001.zip › Table S3.pdf]

**TABLE S3**

## I) Recurrent dreams referred from adolescence to the third therapeutic session

A) In childhood and adolescence:

1) "I follow a downhill street in Sorrento (my town of origin near the sea), that arrives to the sea. I stop in a square from where I can see the sea, but I never arrive to touch it".
2) "I was sinking in the mud"
3) "I was in a sort of basement, where I had lived when I was 8-10 years old. Through a small door it was possible to go out and it was a short cut to go toward my house. Nevertheless instead of reaching the exit and stay in a reassuring place, in the sun, for a non identified reason, I remained in the half-dark room of the basement, that was becoming wider and wider and harrowing. I felt the danger coming from unlikely beings, who might grasp me, catch me. After a certain undefined time of this desperate anguish, in the moment that I felt myself grasped by these beings , with a big effort, I was struggling to free myself, and I woke up"
4) "After my mother's death I have dreamt her for many years"
5) "I dreamt often my mother, who died when I was 7 years old; I have dreamt her up to the adolescence"
6) "I was living at the last floor of a building. I jumped from the balcony; then, when I arrived to the lower floors, I circled and I landed slowly. I climbed again the stairs and rang the bell, and my mother opened the door".

B) In adulthood:

1) "I dreamt often the school and that I had to take my exams again".
2) "I needed to do again my military service and that I was loosing 2 years of my life".
3) "I follow a downhill street in Sorrento…..(This dream was done from the same subject, who referred it in the first period of his life, see A.1).
4) "I am on the big and wide staircase of my secondary school, that is located in a big old building near Venezia square in Rome. There were important and severe rooms. Here I enter in a small door, that in real life was not there, to go toward a dark and variegated room, that is becoming bigger and bigger, huge. The feeling is the same of the previous dream, and so was the end and the exit from the dream".
5) "A tsunami is sweeping away and destroying everything. I try to defend myself inside a fortress or over a hill"
6) "I was a young boy at the lower secondary school, and I couldn't succeed to pass my exams; I was anxious that I wasn't up to it"
7) "For years I have dreamt of flying"

C) In the year before AMI:

1) "I often dreamt to be run over by a tsunami".
2) "I follow a downhill street in Sorrento ….(see the dream above A.1)
3) "I dreamt nightmares before the acute myocardial infarction".

D) During the first psychotherapeutic session:

1) "I have always nightmares".

E) During the second individual session:

1) "I continue to have very distressing dreams and nightmares. In particular I am still dreaming the school and that I have to take my exams again".

2) “I have dreamt continuously for a week that I had to complete a work in the building site, where I am working”.

F) <u>During the third individual session</u>:
1)“I dream always situations where I cannot reach a goal”.

**II) Symbols reported by 3 pts that referred non recurring dreams in their childhood and adolescence**

1) I did “very rich and colored dreams until 10 years ago, when I was 20-21 years old”.
2) I dreamt “to go on a moped”
3) I dreamt a lot until when I was 10 years old.

**III) Symbols, actions and state of mind reported in dreams from adolescence to AMI time**
(here arte reported in cursive the symbols that, from a psychodynamic point of view seem to point out a very dreadful psychological difficulty and conflict, whereas are reported in bold symbols, actions and states of mind showing a positive psychological state):

**AA) <u>In childhood and adolescence</u>**:
1) People: *dead mother; dead mother; mother* .
2) Non animated objects: moped
3) Places: square; *mud*; basement; my house.
4) Landscapes: **sea**.
5) Environment: *half-dark room*; **very rich and colored dreams**.
6) Situations: *nightmare*; *danger coming from unlikely beings….*
7) Actions: *I follow a downhill street that arrives to the sea; I can see the sea, but I never arrive to touch it*; *I was sinking in the mud*; *I remained in the half-dark room of the basement; with a big effort, I was struggling to free myself, and I woke up*; to go on a moped; *I was living at the last floor of a building*. I jumped from the balcony; then, when I arrived to the lower floors, I circled and I landed slowly; *I climbed again the stairs and rang the bell, and my mother opened the door*.
8) State of mind: *desperate anguish.*

**BB) <u>In adulthood</u>**:
1) People: my brother, *my dead wife.*
2) Places: *school*, square, *my secondary school*, big old building, important and severe rooms, l*ower secondary school.*
3) Landscapes: **sea**.
4) Situations: *very distressing dreams*, *sometimes nightmares*, *military service*, *nightmare,* **beautiful relaxing and radiant dreams**, *nightmares.*
5) Actions: *I had to take my exams again*; *speaking in a very harrowing way*; I follow a downhill street that arrives to the sea; *I can see the sea, but I never arrive to touch it; to do military service; to loose 2 years of one's life*; to climb on a grassy slope; *stones rolling down against me*; *I enter in a small door*; to go toward a dark and variegated room, that is becoming bigger and bigger, huge; *I try to defend myself inside a fortress or over a hill*; *to fall down*; *I was driving my car and I had an accident*; *a tsunami is sweeping away and destroying everything*; *I was a young boy at the lower secondary school*; *I couldn't succeed to pass my exams*; flying.
6) ”*I was anxious that I wasn’t up to it*”

**CC)** **<u>In the year before AMI</u>**:

1) People: *my father and mother (who were dead in real life)*- *my dead father*- *my grandmother who had died for a liver disease*- *my mother in law, dead when she was 86 years old for a cardiac disease*- *the father of my wife's actual partner, who had died for a cardiac disease*- my son- *father in law, dead for sudden death.*
2) Places: square.
3) Landscapes: **sea**.
4) Environment: *it was raining*.
5) Situations: *distressing dreams*- *nightmares*.
6) Actions: **I follow a downhill street that arrives to the sea-** *I can see the sea, but I never arrive to touch it*- *I had lost my car*- *my father and mother (who were dead in real life) following me*- *to be run over by a tsunami*- *I sow myself reflected in a mirror, and there was the image of my dead father*- *I was falling down an abyss*- *falling off while I was sleeping*- *my grandmother, dead for liver disease, was very fat, and we were having lunch with many relatives*- *my mother in law, dead for a cardiac disease, entering in my house and giving me her hand*- *I accept and I give my hand to my mother in law, dead for a cardiac disease*- *my son called me to tell me that he had had a car accident*- *my father in law, dead for sudden death, invited me to go with him, telling that beyond one can stay very well*.
