# Supplementary material for: Dream Patterns in Patients with Acute Myocardial Infarction: Data from the STEP-IN-AMI Trial
Source: J Clin Med. 2025 Dec 27;15(1):231. doi: 10.3390/jcm15010231 (PMC12786656; doi:10.3390/jcm15010231)
Supplement: Supplementary file 1 [file Table S1.pdf › Table S1.pdf]

TABLE S1 COMPLETE DREAMS REPORTED BY PATIENTS

PATIENTS	Age	Dreams of childhood and adolescence	Dreams of adult life	Dreams in the year before AMI	First individual session	Second individual session	Third individual session	Fourth individual session	Fifth individual session	Sixth individual session	Seventh individual session	Eight individual session	Ninth individual session	Tenth individual session
1) A. A. female 4 individual sessions and 5 group meetings with her husband	65		Very distressing dreams, and sometimes nightmares. After my retirement I dreamt often the school and that I had to take my exams again. Sometimes I dreamt my brother, and we were speaking in a very harrowing way.	In one of my distressing dream before AMI, "it was raining and I had lost my car". Before the AMI "I dreamt my father and mother (who were dead in real life), and they were following me". I felt this dream as a negative premonition, as if something bad was going to happen.		I continues to have "very distressing dreams and nightmares. In particular I am still dreaming the school and that I have to take my exams again".	Distressing dreams and nightmares finished. "I dreamt my brother, and we were speaking very calmly", whereas in the past we were speaking during the dream in a very harrowing way.	"I was running away, chased by someone that I couldn't identify" . LAST INDIVIDUAL SESSION						
2) G. A. male 4 individual sessions and 5 group meetings with his wife	61		After my military service I had "the recurrent dream that I needed to do again my military service and that I was loosing 2 years of my life". In the last ten years I haven't remembered dreams anymore				" I was with other Italian people at Bracciano (the town where I live), some of them were from Bracciano. We left with a big ship to go to work in America, to do manual jobs as carpentry, etc. In this way we can revolutionize the American society. At last we come back to Italy, and I thought: - What will happen now that everything will fall into politicians' hands?-"	LAST INDIVIDUAL SESSION						
3) A. A. male 8 individual sessions and 4 group meetings with his wife	57								"I was at Anguillara (my town that is on a lake), There is a dark blue sea, all the landscape is dark blue, I am not in the water. At a certain point some friends appear. I cannot see them. They threw a closed mussels necklace to me"			LAST INDIVIDUAL SESSION		
4) D. A. male 6 individual sessions and 5 group sessions with his wife	52			During the months before the AMI "I often dreamt to be run over by a tsunami". Soon before the AMI I had this dream: " I sow myself reflected in a mirror, and there was the image of my father. As my father was dead, I thought that I was going to die, but not for a gastritis (that was my major concern)".				3 dreams: 1) "I lied on the bed with my mother, who is 83 years old. She is a cardiac patient, already operated on of a coronary artery bypass grafting. She was giving off a yellow liquid from her mouth". 2) "I sow Pope Giovanni Paolo 2nd, who was giving his hand to me". 3) "There was a beautiful half-naked girl, who was wearing only panties. I kissed her all up to her pubis".		"I was in Belgium. I see a beautiful church with some icons. There is a kind of dismantled pedalo with oars. I assembled it , and I started rowing on a stormy sea". LAST INDIVIDUAL SESSION				
5) F. A. male 4 individual sessions and 5 group sessions with his wife	62			Dream done soon before the AMI: " I was dreaming that I was falling down an abyss".				LAST INDIVIDUAL SESSION						
6) M. B. male 9 individual sessions and 5 group meeting with his wife	62							"I was working on a scaffolding; I slipped and risked to fall down in an empty space. I called out loudly and asked for help to my brother, who ran to help me"			"I dreamt that my wife was telling me what she had dreamt, and I was telling her what I had dreamt"		LAST INDIVIDUA L SESSION	
7) M. B. male 7 individual sessions and 5 group meetings without his wife	56					He sow in a spontaneous visualization during body relaxation, "a photo taken by my daughter on the lake shore"				Spontaneous vision of "a flash of colored lights, a house, a fireplace, a starry sky"	LAST INDIVIDUAL SESSION			
8) A. B. male 5 individual sessions and 5 group meetings without his wife	70					During an exercise of body relaxation: "I am feeling my body and I have seen a shadow, that is my own image, watching at me from all the sides"	"I dreamt my mother in law, dead 10 years ago"		During an exercise of body relaxation: "I can see my little grandson taken in his mother's arms" LAST INDIVIDUAL SESSION					
9) L. B. male 6 individual sessions and 5 group meetings with his wife	49									LAST IMNDIVIDUAL SESSION				
10) P. B. male 6 individual sessions and 5 group sessions with his female partner	52	A dream repeatedly done since I was 14 years old: "I follow a downhill street in Sorrento (my town of origin near the sea), that arrives to the sea. I stop in a square from where I can see the sea, but I never arrive to touch it". This dream recurred until few months before the AMI	A dream repeatedly done since he was 14 years old: "I follow a downhill street in Sorrento (my town of origin near the sea), that arrives to the sea. I stop in a square from where I can see the sea, but I never arrive to touch it". This dream recurred until few months before the AMI	A dream repeatedly done since he was 14 years old: "I follow a downhill street in Sorrento (my town of origin near the sea), that arrives to the sea. I stop in a square from where I can see the sea, but I never arrive to touch it". This dream recurred until few months before the AMI				"Some little dogs arrive to my house; my children are happy"	Two similar dreams: "I want to do more in the workplace, often replacing other colleagues, also when they do not desire this, and trying to obtain a social consensus"	LAST IMNDIVIDUAL SESSION				
11) M. C. male 7 individual sessions, 1 couple session with his wife, and 5 group meetings without his wife	65								Two dreams: 1) "I am in a cemetery, that is completely flooded; I can save myself climbing on the roof of a car". 2) "My next-door shop neighbor, who was dead for a tumor". (In the real life my neighbor had had a conflict with his wife, who died of a tumor, and their shop was inherited by their children)	Three dreams: 1) "I dreamt my father's brother. He was a delinquent and had been imprisoned in real life, whereas in the dream he was dressed as a policeman". 2) "I was on a locomotive, that was running fast through a steep slope, deep and rumbling gorge; around there were many trees, a chestnut tree, fir trees; in the middle of the trees some ruins". 3) "I am with the animals of my shop on the see shore. A wave arrives and swamped everything. After that some islets remain on a blu and calm sea".	LAST IN DIVIDUAL SESSION			
12) G. C. male 7 individual sessions and 5 group meetings with his wife	53								"I am walking on a road going uphill, but I am not so courageous to look further on"		Two dreams: 1) "I am talking to a serviceman, and I say to him that 120 days have passed, and he answers: 'yes 4 months'." (We calculated together that really 4 months had passed since the acute myocardial infarction and the contemporary beginning of psychotherapy). 2) "I meet a person who keeps a brown dog on a tight rein. I see another dog coming along the street, and he tries to attack me. I find a pillow and I try to defend myself. The dog attacks me again and grabs me with his leg across the pillow". After this I wake up and I wake my wife too. LAST INDIVIDUAL SESSION			
13) L. C. male 5 individual sessions and 5 group meetings with his female partner	56		Dream done when I was 20 years old: "I dreamt to climb on a grassy slope, and some stones rolled down against me". After that dream I didn't dream any more.						For the first time after my twenties I can remember a dream: "I was going to a bar and I met a friend of mine; he was very tall and robust build. He accused me of something, and I defended myself. After that I went out of the bar, and I couldn't find my car" . LAST INDIVIDUAL SESSION					
14) R. C. male 6 individual sessions and 5 group meetings without a partner	50									I haven't remembered dreams for the last 30 years. Today I remember a dream for the first time: "My daughter was taking a coca-cola from the refrigerator, and I told her to drink all the can". LAST INDIVIDUAL SESSION				
15) N. C. male 5 individual meetings and 5 group meetings with his wife	54						"I dreamt a friend of mine, who is an accountant and my son's godfather. He was going bankrupt"		"I had a nightmare related to a person, who has a debt with me of 350 thousands euros". LAST INDIVIDUAL SESSION					
16) G. C. male 4 individual sessions and 5 group meetings with his wife	54						a) "I am with my wife in intimacy in our bedroom. The door is half-closed. My son is passing to go to the bathroom and he sees us through the door" (This really happened when my son was 12 years old, but we parents never had the courage to talk about this event with our child)	"There is my head clerk of two years ago, when I was working in TELECOM. He says to me to give something to eat to the animals in a farm (a cow and others), but I forget to do this" . LAST INDIVIDUAL SESSION						
17) W. C. male 3 individual sessions and 5 group meetings with his wife	48			For 3 months before the AMI I had snored during the night, and I had the "feeling of falling off during the sleep"			2 dreams: 1) "I was going back to my work, and I found again my colleagues and my friends; it was a positive situation". 2) "I found myself again in the same situation, when the acute myocardial infarction happened. I was dressed for a hunting party, but I couldn't see the place, and everything was dark (also if in the reality that place is very beautiful)". LAST INDIVIDUAL SESSION							
18) M. D. male 6 individual sessions and 5 group meetings with his wife	57									LAST INDIVIDUAL SESSION				
19) M. D. male 4 individual sessions	42	I dreamt a lot until when I was 10 years old		Last dream 5 months before the AMI: "I dreamt my grandmother, on part of my father, who had died for a liver disease when I was ten years old. In the dream she was very fat and we were having lunch with many relatives"				LAST INDIVIDUAL SESSION						
20) S. D. male 8 individual sessions and 5 group meetings with his wife	50								"I am driving my car in the traffic. There is a car parked in double line; I overtake it, paying attention to the cars coming from the opposite direction"	"I visualized the container of my two credit cards, and it was empty"		LAST INDIVIDUAL SESSION		
21) M. D. male 6 individual sessions and 5 group meetings with his female partner	61								"I sow my father; he was all black, and he was a mummy without eyes. We were in a port on a big sailing boat, that was all black. There were some small tables and chairs, all empty. My father turns toward me and says to me that we have to start a new refreshment place. He has empty eyes"	LAST INDIVIDUAL SESSION				
22) P. D. male 5 individual sessions and 5 group meetings without his wife	55	I remember only one recurring dream, that I dreamt from when I was 10 years old until adolescence: "I was sinking in the mud"					"I was into a castle; there was a female presence; it was a nightmare and I couldn't go out. While trying to go out, I woke up"	I noted down 3 dreams, but I cannot find them	The nightmares have stopped. Now I have dreams, where I can always reach the aim. "I was at the railway station; there was much crowd; I was looking for my 38 year old nephew, and I found him". LAST INDIVIDUAL SESSION					
23) A. G. male 6 individual sessions and 5 group sessions with his wife	56	From when I was 16 years old until my twenties, I did a recurring dream , a nightmare: "I was in a sort of basement, where I had lived when I was 8-10 years old. Through a small door it was possible to go out and it was a short cut to go toward my house. Nevertheless instead of reaching the exit and stay in a reassuring place, in the sun, for a non identified reason, I remained in the half-dark room of the basement, that was becoming wider and wider and harrowing. I felt the danger coming from unlikely beings, who might grasp me, catch me. After a certain undefined time of this desperate anguish, in the moment that I felt myself grasped by these beings , with a big effort, I was struggling to free myself, and I woke up"	Since when I was 20 to 25 years old I had a recurring nightmare: "I am on the big and wide staircase of my secondary school, that is located in a big old building near Venezia square in Rome. There were important and severe rooms. Here I enter in a small door, that in real life was not there, to go toward a dark and variegated room, that is becoming bigger and bigger, huge. The feeling is the same of the previous dream, and so was the end and the exit from the dream". There have been also beautiful, relaxing and radiant dreams until when I was forty, but I don't remember the details. Since then I don't do dreams, that "let me remind them".	Dream done 2 months before the AMI: "I dreamt my mother in law, dead when she was 86 years old for a cardiac disease. She was entering in my house and she wants to give me her hand. At the beginning I refuse, because I thought that she was dead; but finally I accept and I give her my hand. In the house there was my wife too"						LAST INDIVIDUAL SESSION				
24) S. G. male 5 individual meeting and 5 group meetings with his wife	54	.							LAST INDIVIDUAL SESSION					
25) G. I. male 5 individual sessions and 5 group meetings with his wife	59		A recurring dream of the last ten years: "A tsunami is sweeping away and destroying everything. I try to defend myself inside a fortress or over a hill"						LAST INDIVIDUAL SESSION					
26) F. I. male 8 individual meetings and 5 group meetings without his wife (they LEFT before the AMI)	58			"I dreamt the father of my wife's actual partner. I had known him and he had died for a cardiac disease when he was 75 years old"		"I sow knives in dream"	"My wife was nude and was rubbing cream into her previous employer's penis; their job relationship dated back to 20 years ago and her employer is now 75 years old. This man was a womanizer and in the dream he is nude with his body upside down. I was passing two, three times, and I was asking my wife what was she doing. She answered me that she had to rub cream on his penis. I gave three strong slaps on my wife's bottom"		"I am walking with you (the psychotherapist) through greenery, there is a small lake, and there is peace. I kiss you, but soon after I wake up, because I am afraid of being disrespectful to you"			LAST INDIVIDUAL SESSION		
27) P. L. male 6 individual sessions and 5 group meetings with his wife	46						"I am in a cemetery; it is dark. Smoke is coming out from a grave. My father's body is burning. I extinguish the fire and try to recompose my father's body"			LAST INDIVIDUAL SESSION				
28) R. L. male 7 individual sessions and 4 group meetings with his wife	55					"I have dreamt a very nice and open-minded girl, whom I had known 20 days before"			"I dreamt a lady, the wife of a Cardiologist. I had had a long love affair with her about 10 years ago". I did another dream, that I don't remember		LAST INDIVIDUAL SESSION			
29) C. M. fgemale 9 individual sessions	48								"I am on a shaky staircase, that I must climb to reach the attic room, where my friend Claudia lives. I hold a child's hand and I arrive into the attic room with hard physical exertion. From the balcony I see Claudia, who was below, in a courtyard near a big walnut tree. Then I, Claudia and the kid leant against the balcony railing, that breaks, and we fall down in the courtyard. We hold on to the broken railing, and we succeed in falling down on our feet without hurting ourselves"		" I am with a little girl, who cries in despair. There is also a colleague of mine, who I am preparing with for the certificate of hospital coordinator (in real life I don't know her). We wander around Rome, and we enter in a cemetery, because my grandmother had died (my grandmother had died when I had started working as a nurse). There are graves with tombstones of typical from Lecce stone (my native city), and many monitors with electric wires. There is also a staircase, that goes in a ravine, where it is possible to eat food, but I cannot eat. We go out by a car, and my friend is driving. We are wandering again in Rome streets. There is a cemetery on a building roof. The little girl continues crying"		LAST INDIVIDUA L SESSION	
30) C. M. male 7 individual sessions and 5 group meetings with his wife	67		Recurrent dream up to some years ago: "I was a young boy at the lower secondary school, and I couldn't succeed to pass my exams; I was anxious that I wasn't up to it"	Dream of one month before the AMI: "My son called me to tell me that he had had a car accident"				"I was a young man, and I was with a film director, who I had worked with some years ago for a film shotted in the desert. In real life this director had died when he was 59 years old, and in the dream I felt the same anguish that I had felt during the film shooting"			LAST INDIVIDUAL SESSION			
31) G. N. male 5 individual sessions and 5 group meetings with his wife	43	"After my mother's death I have dreamt her for many years"				During an exercise of body relaxation: "I visualize my two children"	Two dreams: 1) "I was with my brother, and we had a colored bandanna on our head. We were cooking; my brother was cooking the first course , whereas I was cooking barbecued meat". 2) "There was my mother, who was helping us with the two children" .	"I have dreamt my friends of Milan, who I am meeting sometimes, also if sometimes we can meet after a long interval, more than one year"	LAST INDIVIDUAL SESSION					
32) C. N. male 2 individual meetings and 5 group meetings with his wife	31	I did "very rich and colored dreams until 10 years ago, when I was 20-21 years old"	"Since I was 20 recurring dreams have started, where I dreamt to fall down". Last dream that I can remember is of some years ago: "I was driving my car and I had an accident"			Two dreams of this week: 1) "I was dressed with a jeans suit, and I was playing football in my house garden with two longstanding friends of mine. One of them is old as I am, and graduated in economics 3 years and half ago, but since then he is not working. The other friend was the son of a hospital employee, who is going to retire. This friend in an accountant, and he does only this in his life". 2) "I attend a game between Rome and Juventus (two Italian football teams), that had been played before I had the acute myocardial infarction. I am a Rome supporter, and in the dream the Juventus team wins the game". LAST INDIVIDUAL SESSION								
33) E. P. male 4 individuale sessions and 3 group meetings without his wife	53					"I dreamt twice to smoke a sigarette"		LAST INDIVIDUAL SESSION						
34) M. P. male 5 individual sessions and 5 group meetings with his wife	50					Two dreams: 1) "I have dreamt continuously for a week that I had to complete a work in the building site, where I am working". 2) "I was smoking a pack of cigarettes".	"I dreamt my ex building site boss and the my working place".	Two dreams. 1) "I couldn't have the hunting license". 2) "There were many wild boars, and I couldn't shoot anyone".	"My father was alive (in real life he died 10 years ago), and he was coming with my brother and offered me a lot of things to eat". LAST INDIVIDUAL SESSION					
35) G. P. male 5 individual sessions	70		I have had nightmares since when I was very young	I dreamt nightmares before the acute myocardial infarction	Again I have always nightmares. The last one was soon after the primary PTCA for the AMI: "I dreamt that a scaffolding was falling on top of me"		"A tree fell down on my bed"		LAST INDIVIDUAL SESSION					
36) A. P. male 5 individual sessions and 5 group meetings with his wife	56	"When I was a young boy I dreamt to go on a moped, because my parents prohibited me to go"							LAST INDIVIDUAL SESSION					
37) A. P. male 4 individual sessions and 5 group meetings without a partner	36						"I took a small child in my arms; he was very beautiful, and I didn't know how to do".	LAST INDIVIDUAL SESSION						
38) F. P. male 8 individual sessions and 5 group meetings with his wife	57	"I dreamt often my mother, who died when I was 7 years old; I have dreamt her up to the adolescence"						"I was behind a bookcase, and I threw books on a person, but I cannot say who that person was"		1) "I am looking a brown running horse, that is ridden by my family female doctor; she is turned on the contrary, toward the bottom of the horse, and she holds in front of herself her 2 year old daughter". 2) "A woman with fishnet tights, that are similar to the net put around the roast beef. I put a basil bunch under the net". During a relaxation technique this visualization: "I was in a green valley; the sky was blue; a herd of multicolored horses were in the paddock".	"I was driving a car on a bend mountain street. I goes out of the street and I start flying on a green valley". During a relaxation technique: " I see the Maldive islands landscape, the blue sea; the smells and fragrances"	During a relaxation technique: "I see the Alps". LAST INDIVIDUAL SESSION		
39) M. P. male 5 individual sessions and 5 group meetings. His wife participated to one group meeting	62								"I was trying to transfer some decisions to a collaborator of mine. My collaborator answered me that it was okay if he had already decided". LAST INDIVIDUAL SESSION					
40) A. R. male 10 individual sessions and 5 group sessions; his wife participated to 3 group sessions	51								"I go to a fair with my wife. It is dark. Then my wife goes away with a male doll"		"I am with my brother, who is guiding me. I enter in a tunnel and I emerge into a castle. There are many meadows and green places, but there isn't a way out, and I get always in a street, that takes to a ravine"	"I am on a ship; there are many tidy rooms, things to eat"		"I am in a garage. There is a stair, that goes into the garage. The floor is covered of little stones. I have to pave it all. From the garage leaves a street that takes out. I have to tidy up the entrance of the garage, so that water cannot enter. In fact water flows out, without doing a marsh and without entering into the garage". LAST INDIVIDUAL SESSION
41) M. R. male 4 individual sessions and 5 group meetings with his wife	56	A recurring dream since when I was 5 years old up to 15 years old: "I was living at the last floor of a building. I jumped from the balcony; then, when I arrived to the lower floors, I circled and I landed slowly. I climbed again the stairs and rang the bell, and my mother opened the door". Since I was 15 I haven't remembered dreams for many years	"Seldom I have dreamt my dead wife, and when I dreamt her I sow a vague face"					LAST INDIVIDUAL SESSION						
42) F. R. male 5 individual sessions and 5 group meetings with his wife	54								LAST INDIVIDUAL SESSION					
43) M. S. male 6 individual sessions and 5 group meetings without his wife	53									LAST INDIVIDUAL SESSION				
44) M. S. male 5 individual sessions and 5 group meetings with his wife	56						"I dream always situations where I cannot reach a goal"		LAST INDIVIDUAL SESSION					
45) R. T. male 6 individual sessions and 4 group meetings with his wife	56						"I am driving a motorcycle toward a high place like Zodiaco (that is an high hill in Rome). At a certain point the motorcycle broke and stopped. A wire that goes to the carburetor broke. I try to repair it, without success. After, I am with a little 8 year girl; she is my daughter with her dog. I say that we have to send away the dog, and I am sorry"			LAST INDIVIDUAL SESSION				
46) D. V. female 7 individual sessions and 5 group meetings with her husband	59			"I dreamt my father in law, dead last September for sudden death. He was very well, and invited me to go with him, telling that beyond one can stay very well"	Dream soon after the AMI: "I was on a reef; in front of me there was the sea, that was like a pond. A hand pushed me and I fell in the water. I went to the bottom and I couldn't go up. I felt the water weight, and I felt that I was drowning"			"I dreamt my second daughter's partner, who was arrested by the police. The policeman showed me the identity card of the arrested boy, and there was written 'ROM'. I answered that I was right, he was a ROM, a gypsy". Other fragments of dreams are related to "my job, and my first daughter, who has been kept back in Egypt"			LAST INDIVIDUAL SESSION			
47) G. F. male 4 individual meetings and 5 group meetings without his wife	50		"For years I have dreamt of flying"					LAST INDIVIDUAL SESSION						

Childhood: period from infancy to the age of 11.
Adolescence: period approximately from the age of 12 to 20.
Adult life: period from the age of 21 to the beginning of the year before AMI
AMI: acute myocardial infarction
